# Review of Policy Framework for the Development of Carbon Capture, Utilization and Storage in China

**DOI:** 10.3390/ijerph192416853

**Published:** 2022-12-15

**Authors:** Dongdong Song, Tong Jiang, Chuanping Rao

**Affiliations:** 1 School of Public Administration, China University of Geosciences (Wuhan), Wuhan 430074, China; 2 Key Laboratory of Ministry of Natural Resources for Rule of Law Study, Wuhan 430074, China; 3 Data Law Research Institute, Jiangxi University of Finance and Economics, Nanchang 330013, China

**Keywords:** carbon capture, utilization and storage (CCUS), policy, law

## Abstract

Carbon capture, utilization and storage (CCUS) has been applied in many countries and has proven to be a key carbon-reduction technology for the future. China currently emits the most carbon, and prior research findings indicate the high potential of CCUS technology to support the country’s emission-reduction process. China introduced CCUS technology at the end of the 20th century and has since implemented a series of related policies. This paper compares the development status of CCUS in China and other countries, studies the legal and policy framework and the development process of CCUS in China, and analyzes the defects in relevant laws and policies. The results show that China’s current legal and policy system is not conducive to the further development of CCUS; specifically, there is no special law, and the policy system is incomplete. Consequently, it is difficult to advance and give full play to the emission-reduction effect of CCUS. To promote CCUS development in China, this paper proposes corresponding countermeasures, including formulating a special law, perfecting the CCUS policy system, expanding government financial support, and improving CCUS public awareness and support.

## 1. Introduction

Global warming, caused by emissions of a large number of greenhouse gases into the atmosphere, is among the most difficult challenges for humans to solve. CO_2_ is the main greenhouse gas emitted by human activity. Protein malfunctions in cells due to elevated CO_2_ and associated low pH have the potential to cause threats to life, including cancer, neurological disorders, lung disease, diabetes, etc. [1]. There is now substantial evidence that permanent exposure to CO_2_ levels predicted in the future will have significant effects on humans [2].In 2018, the Intergovernmental Panel on Climate Change issued a special report on global warming, warning that the global temperature rise should be limited to 1.5 degrees Celsius [3]. In 2019, China accounted for over one-quarter of the world’s carbon emissions, reaching 13.7 billion tons of CO_2_ equivalent [4]. At the Leaders’ Summit on Climate in 2021, President Xi Jinping expressed China’s determination to reduce emissions by proposing clear emission-reduction targets: to achieve a carbon peak by 2030 and carbon neutrality by 2060.

Carbon capture, utilization and storage (CCUS) has attracted much international attention. This technology is proven to be the most important means of reducing CO_2_ produced by burning fossil energy [5]. In recent years, demonstration and application studies of CCUS projects have been conducted in China and elsewhere [6]. CCUS is divided into four main steps [7]: CO_2_ capture, transportation, utilization and sequestration. First, CO_2_ is mainly captured from the gases generated from fossil fuel combustion or from the gases produced by intensive, high-emission manufacturing industries (such as cement and petrochemical plants). The capture efficiency is generally above 90% and can even reach 100% in operational projects. Second, after CO_2_ is captured, it is transported to the storage site, generally by pipeline (most common) or vehicle. Third, captured CO_2_ is then sequestered in suitable geological layers, where it can be stored for hundreds or even thousands of years. Forth, captured CO_2_ plays an important role in industrial production, and it is well-accepted as the most affordable and abundant nontoxic C1 source [8].

At present, CCUS is mainly applied in large coal-fired power plants and in the petrochemical, cement-processing, and steel industries. Captured CO_2_ is mostly reused. Injecting captured CO_2_ into oil fields to improve the oil recovery rate and storing CO_2_ in oil fields is a common utilization method. The International Energy Agency (IEA) predicts that CCUS will contribute 14–19% of emission reductions in 2050 if emissions are stabilized at half of the 2005 levels [9]. In recent years, China has been vigorously developing renewable energy, achieving remarkable results. However, many studies point out that China will still rely on fossil energy in the future [10,11]. If no countermeasures are implemented, the consumption of fossil energy will still produce vast CO_2_ emissions. Therefore, the Chinese government needs to deploy more CCUS projects in the future to reduce the CO_2_ produced by consuming fossil energy.

## 2. Literature Review

China’s industrialization lags behind that of developed countries, led by the United States, and the development of CCUS started later than those countries. Although the technology has developed rapidly, CCUS has not been industrialized and commercialized in the country, and even large-scale complete demonstration projects have not been completed. Thus, the development of the CCUS industry has been seriously hindered. Huang believes that a CCUS legal system should be established in China that reflects macro and micro perspectives: a special law should be formulated on CCUS, supported by a regulatory system encompassing accountability, emergency response, information disclosure, and public participation [12]. In an empirical study of a CCUS project in Ordos, Yu et al. identified problems such as lack of market access and regulatory standards, no guarantee of stakeholders’ rights, lack of legal liability provisions, and insufficient incentivization of market financing. Proposed solutions include the formulation of laws to comprehensively regulate CCUS-related activities [13]. Peng et al. also recognize that there are no relevant laws and regulations governing the use of CCUS technology in China. Since this technology entails high risks, it must be regulated by administrative departments of the government. He also contends that CO_2_ should be legally regulated as a pollutant in CCUS legislation [14]. Based on a systematic review of CCUS-related policies and regulations of key countries and regions under international conventions, as well as China’s legal system and CCUS policies and regulations, Huang et al. contend that the following key points should be addressed in China’s CCUS legislation: identification of CO_2_; determination of surface rights and underground rights; protection of health, safety, and environment; transfer and protection of intellectual property rights; a project approval system; and establishment of an incentive policy system [15]. These findings indicate that the core impediments to developing the CCUS industry in China are the lack of laws, imperfect policies, and limited public awareness. Aiming to solve these problems, this paper compares the development of CCUS in China and abroad, analyzes the current legal and policy framework and development process of CCUS in China, discusses the characteristics and shortcomings of existing laws and policies, and proposes corresponding countermeasures.

## 3. Global Development Status of CCUS

The pace of development and deployment of CCUS facilities has continued to accelerate worldwide in recent years, with the number of large CCUS facilities growing to 51 by 2019 [16]. Among these, 19 are in operation, 4 are under construction, 10 are in advanced development, and 18 are in early development. Facilities currently in operation or under construction are capable of capturing and permanently storing approximately 40 million tons of CO_2_ per year. Large CCUS facilities are mainly concentrated in North America. Table 1 shows the large CCUS projects (annual CO_2_ capture over 1 million tons) currently in operation in the world [17].

The CCUS project at the Barrow Island gas processing plant off the coast of Western Australia began injecting CO_2_ in August 2019. It is the world’s largest geological CO_2_ sequestration facility, with a capacity of up to 4 million tons per year [18]. The Shute Creek gas processing plant in Wyoming (USA) captured over 100 million tons of CO_2_ from its operation until 2019 and uses captured carbon to enhance oil recovery [19].

In terms of cost, CCUS has a large capital demand and needs much more financing to enable industrial development. The IEA estimated that 100 CCUS projects would need to be developed globally by 2020, requiring an additional USD 54 billion in investment in addition to government investment. By 2050, 3400 projects need to be developed globally, which will require additional investment of USD 25,000–3 trillion [16]. The International Energy Agency (IEA) estimated that China and India would need USD 19 billion to develop CCUS projects between 2010 and 2020 and USD 1.17 trillion in total between 2010 and 2050 [20].

## 4. Development Status of CCUS in China

### 4.1. Operation Status of CCUS Project in China

In recent years, China has yet to complete megaton CCUS projects to be built in operating catches. The biggest project is the CCU demonstration project for the Shengli Oilfield, located in the Shandong Province. The carbon yield is 400,000 tons per year, and the capture of CO_2_ is used for the injection of oil into the field or oil displacement (EOR, enhanced oil recovery). Table 2 shows CCUS projects currently operating in China with an annual capture of over 100,000 tons [21]. In 2007, the CNPC Jilin Oilfield CO_2_-EOR Research and Demonstration project realized the industrialization of CCUS-EOR technology for the first time in China, representing a milestone CCUS project in China. Subsequently, the Shenhua Ordos new coal-to-oil CO_2_ capture and storage project in 2011 was China’s first test project of saline geological storage [21].

In general, China’s carbon capture is mainly distributed in the north, and most application scenarios are coal-fired power plants. Some high-emission chemical industries have also carried out experiments, and most of the CO_2_ captured by the chemical industries is used for oil displacement. There are currently two ways to reuse captured CO_2_ in China: CO_2_-enhanced petroleum exploitation technology and CO_2_-enhanced coalbed methane technology. Both are of great significance to China’s energy strategy and can respectively help China achieve stable oil production and improve the exploitation and utilization of coalbed methane. China’s national major technology research project, “greenhouse gases to improve recovery efficiency of resource utilization and underground storage”, has experts from China’s Ministry of Land point out that within the scope of geologic reserves, about 13 billion tons of crude oil is suitable for CO_2_ flooding technology and can be improved by about 15% compared to the original recovery rate; therefore, the oil production of 1.92 billion tons can be increased, and at the same time, oil fields can also sequester about 5 billion tons of CO_2_, so the application of CCUS technology plays a certain role in promoting China’s crude oil and mining industry [23].

### 4.2. Current Situation of CCUS Deployment Potential in China

**Figure 1:**
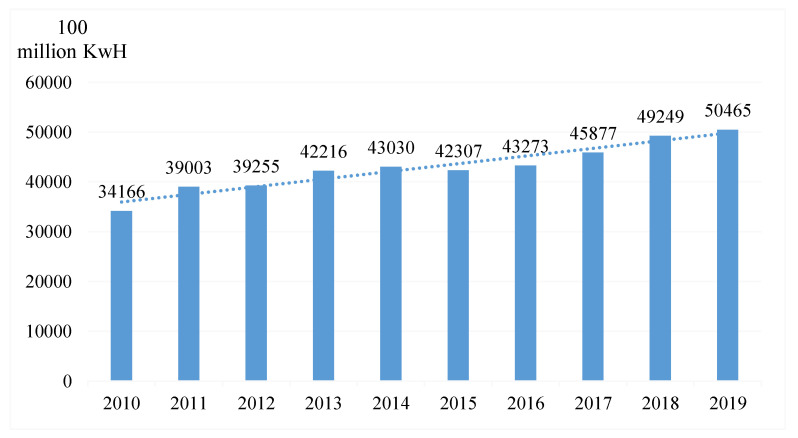
China’s annual thermal power generation capacity, 2010–2019 [28].

China is now the world’s largest generator of electricity, producing 25% of the global total [24]. However, China’s power supply remains dominated by fossil energy consumption, resulting in extraordinarily high annual CO_2_ emissions from its thermal power plants. These plants account for the highest proportion of China’s total annual carbon emissions. The Chinese government aims to help solve the problem, on the one hand, through the technological breakthrough of fossil energy use efficiency, as this method can reduce the consumption of fossil energy obviously in the short-term, but in the long-term, because of the limitation of technical bottlenecks, after reaching a certain value of fossil energy, use efficiency is difficult to ascend, or ascending costs will outweigh the benefits. The government has also increased public investment in renewable energy, especially clean energy, such as hydro, photovoltaic, and wind power, which have increased sharply in recent years. Consequently, the share of power generated from renewable energy sources has also increased year by year. By 2019, 17.8% of hydropower and 8.6% of wind and photovoltaics power generation in China were generated. However, with numerous studies suggesting that China will continue relying on fossil fuels for power, CCUS technology is key to achieving large-scale sustainable use of these fuels [25]. CO_2_ produced by coal-fired power plants is one of the main sources of greenhouse gases in China every year, and these emissions are characterized by large volume and concentration, which is one of the main scenarios for CCUS application. To achieve the government’s goals of carbon peak and carbon neutrality, it will be necessary to apply CCUS technology in coal-fired power plants [26]. The vast majority of China’s power plants are coal-fired, and with the coal-dominated energy mix unlikely to change soon, China is likely to continue building coal-fired plants to meet the pressure to reduce emissions from its growing power demand [27]. Although the proportion of thermal power generation in China is decreasing year by year, only accounting for about 70%, its total amount is increasing, as shown in Figure 1 [28].

In Figure 1, it is clear that China had a steadily rising trend of thermal power electricity for nearly a decade. The Chinese government plans to realize the goal of carbon peak in 2030. The economy is still in the next 10 years projected to pursue sustained growth, the demand for electricity is still growing, and China’s power-generating capacity in the next period of time will continue to rise. In 2020, energy consumption will account for about 85% of China’s total CO_2_ emissions and 70% of its total greenhouse gas emissions [29]. Wei et al. analyzed the carbon-emission-reduction potential of CCUS affiliated with the National Energy Group (formerly Shenhua) through the screening method of coal-fired power plants, the screening method of CO_2_ geological storage sites, the technical and economic evaluation method of the whole process of CCUS, and the source and sink matching method. More than 60% of the 72,720 MW units of the former Shenhua Coal-Fired Power Plant have the basic conditions for CCUS transformation [30]. Fan et al. found that 30 out of 441 Chinese counties have CCUS and emission-reduction potential, with a total of 99.01 million tons/year, and the total emission-reduction of CCUS in the top five provinces—Hebei, Xinjiang, Tianjin, Jiangsu, and Anhui—accounted for 83.9%. These and other provinces can serve as demonstrations for the deployment of CCUS projects in the near future [31].

### 4.3. Technical Cost Analysis of CCUS in China

**Figure 2:**
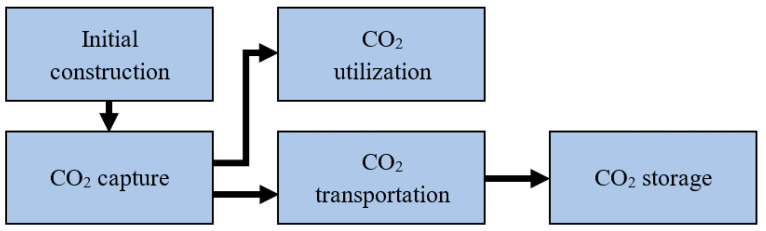
Cost elements of CCUS.

The main cost elements of CCUS are for initial construction, capture, transportation, and storage, as shown in Figure 2. The initial construction cost of a CCUS project is particularly high. Currently, the costs of deploying equipment with a 90% carbon capture rate in 400 and 800 MW power stations are about USD 70 million and USD 150 million, respectively [32].

After the project becomes operational, the main costs relate to capturing, transportation, and storage. As of 2020, the capture cost is the highest, reaching 300 CNY/ton of CO_2_, while the transportation and storage costs are 1 CNY/ton and 60 CNY/ton, respectively. The cost of carbon capture is forecast to drop to 220 CNY/ton by 2035 and to 135 CNY/ton by 2050, while transportation and storage costs are predicted to drop, respectively, to 0.6 CNY/ton and 40 CNY/ton by 2035 and to 0.45 CNY/ton and 30 CNY/ton by 2050 [33]. Fan et al. used the learning curve model and cost optimization model to study the total cost of commercializing CCUS in China and the transformation potential of CCUS in national and provincial coal-fired power plants. Their results indicate that advancing the commercialization of CCUS to 2030 can greatly reduce the risk of technology lock-in by increasing the CCUS renovation potential to 431.1–499.90 GW at an estimated cost of USD 54.3 billion [34].

In the short to medium term, CCUS cost is the biggest barrier. In the long term, however, CCUS is very cost-effective compared to other mitigation options. The range of cost estimates is wide, depending on the type of process, separation technology, CO_2_ transfer technology, and storage location [35]. Compared with the other three costs, the capture cost is most influenced by technology. Accordingly, most CCUS research worldwide focuses chiefly on the technical requirements for reducing the cost of carbon capture. This cost still exceeds the benefits under the current Chinese government subsidy, which is a major impediment to introducing CCUS technology in China.

### 4.4. Public Perception of CCUS in China

The public’s understanding of CCUS will affect their acceptance of and support for the technology. One obstacle to further deploying CCUS is low public perception and support [36]. Liu et al. developed a behavioral model to predict public acceptance of CCUS using the ABC (affect, behavior, and cognition) model based on attitude analysis. Their findings suggest that public cognition has a significant impact on public acceptance and that perceived gain has a higher indirect impact than perceived risk [37]. Using articles on CCUS technology collected from national, provincial, and municipal newspapers in China, Jiang et al. performed a qualitative and quantitative content analysis to study how CCUS knowledge is transmitted and the media’s attitude toward this knowledge. They also compared CCUS marketing content in domestic and foreign newspapers. Their findings suggest that current news coverage of CCUS in China is insufficient due to technical misunderstandings and lack of comprehensiveness [38]. From the point of current research achievements, the Chinese government on CCUS propaganda dynamics is weak. The Chinese media also ignore detailed reports of CCUS technology, and the lack of details of the whole process of CCUS propaganda means that the public understanding of CCUS is vague. The public will realize more about the technical macroscopic problems, such as the macroeconomic policy concept, rather than micro information, such as technical processes and project presentations.

## 5. Development Status of CCUS Policies and Laws in China

### 5.1. Legal Regime for CCUS

China is currently the world’s largest consumer of coal, and its cement and petrochemical industries produce higher annual CO_2_ levels than any other industry in the world. Despite China generating the highest carbon emissions in the world, it still has no special legislation on CCUS. At present, China’s legal norms, management mechanism, and technical specifications for CCUS project activities are mainly based on existing legal instruments, such as the Civil Code and the Air Pollution Prevention and Control Law.

In the process of CCUS project operation, operators of the whole process, including investment, construction, storage and other participants, are the main actors that will participate in the activities of CCUS, in addition to those based on the legal obligations and contractual obligations, those involved in the operation of the project’s main responsibilities, the main body of CCUS project activities, the basis of the Civil Code and the relevant provisions of contract law. It project is regulated by some existing laws and regulations within the scope of liability and obligations. Generally, the operators should bear responsibility for environmental damage and harm to public health and compensate for loss or damage caused by the implementation of performance and sealing and CO_2_ leakage accidents. However, China’s general principles of civil and contract law are not clear on these aspects for CCUS. Therefore, the scope of responsibility of CCUS participants needs to be identified in existing laws and regulations, and the scope of responsibility of each link should be determined so as to regulate the behavior of CCUS legal subjects. In the Air Pollution Prevention and Control Law, CO_2_ is not treated as an air pollutant. China’s attitude toward CO_2_ is in line with one of the modern international definitions, regarding CO_2_ as a waste product rather than a pollutant. Air pollutants are substances whose direct or indirect discharge into the atmosphere causes pollution. CO_2_ is an important component of the atmosphere, so it is clearly unreasonable to regard it as an air pollutant. According to this, CO_2_ can only be used as the waste generated in modern production. As CO_2_ is both the main greenhouse gas and an important part of the atmosphere, CO_2_ emissions from industrial production should be subject to standardized controls. However, the Air Pollution Prevention Act has no clear laws and regulations on waste CO_2_: this gap needs to be addressed.

### 5.2. CCUS Policy Development

Since 2006, the Chinese government has begun formulating policies related to CCUS. This section analyzes the main development context of CCUS policies in China in five-year periods.

**Figure 3:**
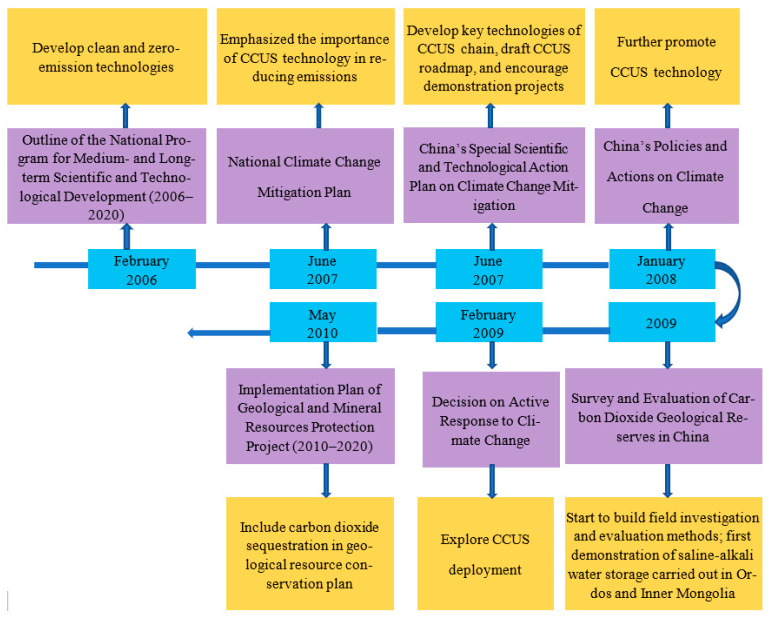
China’s CCUS policy framework for 2006–2010.

The years 2006 to 2010 were the period of China’s 11th Five-Year Plan. Although government policies during these years did not directly mention CCUS technology, they gave some support for CCUS. At this time, China’s CCUS was in the early preparation stage. The government issued the “Outline of the National Medium- and Long-term Program for Scientific and Technological Development (2006–2020)” [39] and “China’s Special Scientific and Technological Action on Climate Change” [40]. The first plan marked the beginning of China’s low-carbon development. Although it did not stipulate specific technologies for reducing carbon emissions, it called for clean and green development. The latter two plans regard CCUS as a key carbon-reduction strategy and define the purpose of developing CCUS technology. Figure 3 shows the framework of CCUS-related policies issued in China from 2006 to 2010.

**Figure 4:**
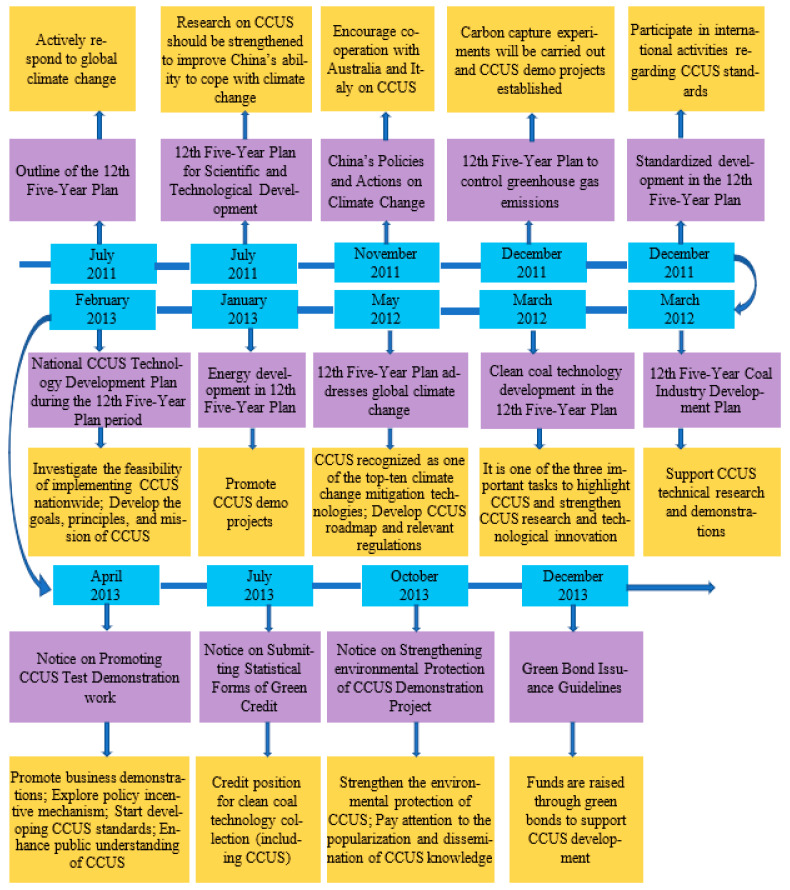
CCUS policy framework for 2011–2015.

During the period of China’s 12th Five-Year Plan (2011 to 2015), amid the growing urgency of global climate change, the Chinese government increasingly highlighted the importance of CCUS as one of three key approaches to clean coal development and one of the ten technologies to mitigate climate change. The government also provided a certain channel for CCUS investment. Figure 4 shows the policy framework of CCUS in China from 2011 to 2015.

**Figure 5:**
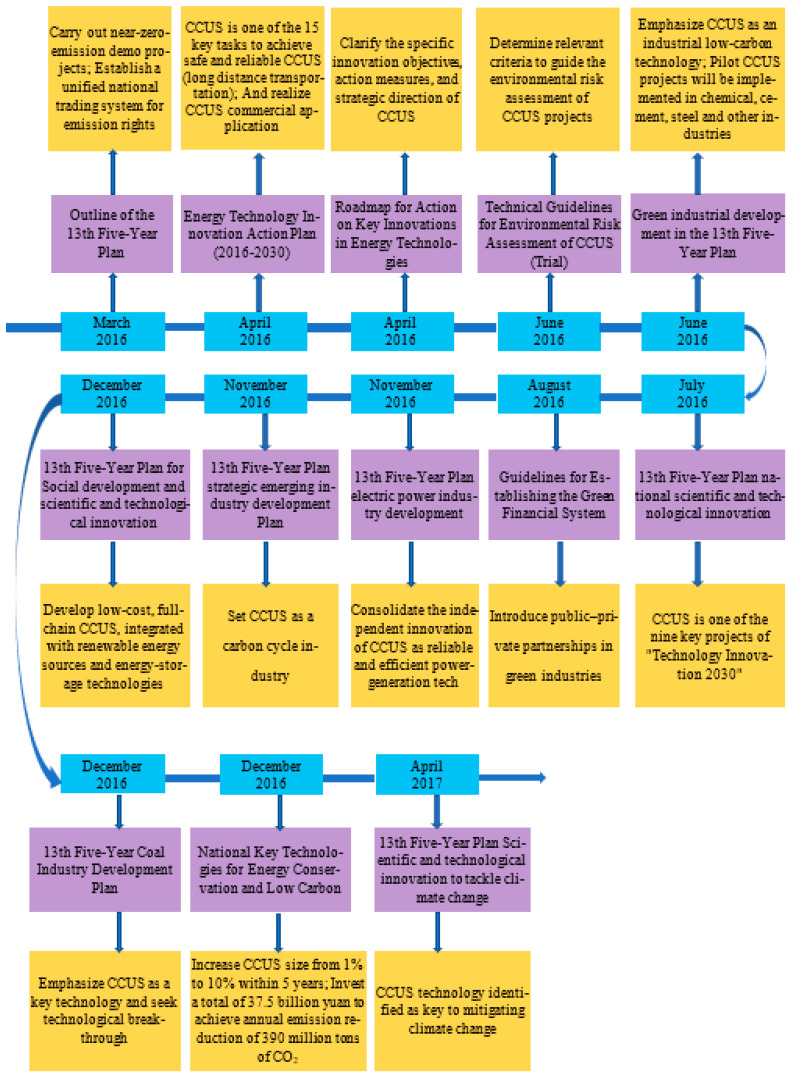
CCUS policy framework for 2016–2020.

From 2016 to 2020, CCUS became more widely covered by various policies formulated under the 13th Five-Year Plan. For example, in the electric power industry, CCUS was rated as a credible green power generation technology and became one of the nine Science and Technology Innovation 2030—Major Projects [41]. In addition, the inclusion of CCUS technology in the “National Key Energy-saving and Low-carbon Technology Promotion Catalogue” has increased the deployment rate of CCUS from 1% to 10% [42]. In terms of financial support, seven government departments have formulated “Guidance on Building a Green Financial System” [43], which aims to adopt a public–private partnership approach to encourage cooperation between state-owned and private enterprises. Many provincial-level policies have also been released. Figure 5 shows the policy framework for CCUS from 2016 to 2020.

In the 14th Five-Year Plan released by the Chinese government in 2021, the goals of carbon peak in 2030 and carbon neutrality in 2060 are set, and the construction of major CCUS demonstration projects and other contents are clearly proposed. This shows a rise in government attention to preventing climate change and increasing awareness of the importance of achieving carbon-reduction targets through CCUS technology.

At the policy level, the State Council issued “China’s National Plan to Address Climate Change” in 2008 [44] and simultaneously set up the Energy Conservation and Emission Reduction Work Leading Group. Subsequently, the 12th Five-Year Plan established CCUS technology development as a national strategy, and from a technical point in the comprehensive planning, proposed the CCUS development objectives and requirements. First of all, across the country, demonstration projects are encouraged to be set up; second in the basic principles is development goals, with a priority on the development direction and key tasks and supporting measures, etc., which all have relevant requirements. In 2013, the National Development and Reform Commission issued the “Notice on Promoting the Experimental Demonstration of Carbon Capture, Utilization and Storage” [45]. In the same year, the government issued the “Notice on Strengthening Environmental Protection of Carbon Capture, Utilization, and Storage Pilot Demonstration Projects” [46]. The notice analyzed the deployment of CCUS projects in terms of the subsidy system, standard system, and international cooperation and proposed to disseminate CCUS technology information to the public and thereby strengthen the public foundation. These two notices are the policy provisions most clearly intended by the Chinese government to convey to the public the attitude toward CCUS technology. However, while the government has been releasing policy documents to promote CCUS development, it must be admitted that CCUS remains largely neglected in China.

### 5.3. Problems in Relevant Laws and Policies on CCUS

#### 5.3.1. CCUS Technology Lacks a Legal System

China has not yet formulated special CCUS regulations, and existing laws and regulations are unsuitable for effectively regulating new technologies, especially in terms of market access and legal supervision. Moreover, China’s current legal framework lacks several key systems, such as subject qualification standards of CCUS, the liability mechanism during project operation, the long-term liability mechanism for storage sites, the legal regulation of CO_2_, and the public participation mechanism [47]. In the process of deploying and implementing CCUS, the absence of these legal systems will produce a series of problems, such as unclear rights and responsibilities of implementing subjects, difficulty in supervising CCUS, and unguaranteed rights of the public. Since the law requires comprehensive factors such as economy, society, production technology, it not only takes a certain amount of time but also has high requirements for the development of legislative technology. Furthermore, a CCUS special legislation is more difficult to achieve in a short period of time, but it must combine the policy system of China that has been formed in recent years and related legal documents. It is still desirable for the government to issue a special law on CCUS as soon as possible. The development of CCUS technology itself must ensure the stable operation of the technology and system before it can be effective. In the process of formulating CCUS-related laws in China, the risks in the system must be clearly identified and effectively avoided.

#### 5.3.2. China’s CCUS Technology Policy Is Inappropriate

Over the past decade, China has made significant progress in the policy framework for CCUS, centering on the gradual development of the national five-year plan on climate change mitigation, energy-related development, and technological innovation. However, these policies only promote CCUS technology at the macro level, which is insufficient. First, most of the policies lack operability, and their practical application rarely produces the expected effects, meaning that local governments are unable to implement these policies. Second, there is too little market investment in the CCUS industry, enterprises lack confidence in the long-term development of CCUS technology (compounded by the weak promotion through policies), and the investment cost of CCUS in the early stage is huge. At present, there is no fiscal or tax support for CCUS technology, capital, and other aspects in China. The CCUS demonstration projects in operation rely primarily on self-financing by state-owned enterprises and support from government funds [48]. Finally, the Chinese government’s marketing of CCUS is obviously insufficient, resulting in the lack of public awareness of CCUS technology and difficulty obtaining public support for its development and industrialization, thus blocking its deployment in China.

## 6. Legislation and Policy Countermeasures to Further the Development of CCUS in China

### 6.1. Establish a Special Law on CCUS

Calls are intensifying for CCUS legislation in China. First, there is no provision on carbon capture and sequestration in China’s current laws. Second, CCUS legislation may represent China’s first climate law. In other countries, regulation of CCUS is generally issued in the form of special issuance, which is comprehensive and systematic standardization, has a high legislative starting point, and has certain forward-looking legislative content worthy of China’s reference. CCUS is connected to politics, people’s livelihoods, and the economy, and its practices should develop concurrently with the formulation of legislation. It cannot and should not await the institutionalization of a comprehensive and perfect legal system. The enactment of special legislation is particularly important for CCUS in its early development stage.

To comprehensively promote and regulate CCUS, legislation should mainly include a market-access system and a regulatory system. As regards market access, given the particularity of the CCUS industry, strict standards and a rigorous inspection regime should be formulated to obtain the subject qualification. Meanwhile, the CCUS regulatory system should be designed to include the following elements:

(a) *Environmental impact assessment or environmental risk assessment*. To obtain project approval, all CCUS projects must carry out environmental impact or risk assessment for the whole project life cycle under the legal framework.
(b) *Storage site selection management*. Mothballed sites must undergo strict risk assessment procedures, and sites with minimal leakage risk and low risk should be selected in accordance with unified international standards.
(c) *Approval*. The approval system includes risk assessment and monitoring, and the approval process should be open and transparent with respect to relevant information disclosure and hearing procedures. The approval system should specify the conditions and procedures for applications and the issuance of permits.
(d) *Monitoring*. CCUS technology and demonstration projects should be monitored long-term and continuously with respect to safety, environmental protection, public health, and resource management. Monitoring methods that are measurable, monitored and verifiable should be adopted.
(e) *Safety*. Unified safety standards should be formulated, and the safety of storage sites, transportation pipelines, and safety risks during transportation should be actively managed.
(f) *Responsibility*. Regulation is particularly needed on the project operator’s responsibilities, including the development of transparent operational and execution plans, monitoring, liability during and after site closure, and reporting of remedial actions.
(g) *Accident emergency handling*. This system needs to include a hazard prevention zone and hazard alarm monitor, CO_2_ leakage remediation measures, emergency reporting procedures, and emergency remediation measures for managers.

In addition, laws and regulations on energy, energy conservation, renewable energy, circular economy, environmental protection, and other related fields should be further revised to promote CCUS as a means to tackle climate change. By maintaining consistency between policies and actions in various fields, valuable synergies can be formed.

### 6.2. Further Improve the CCUS Policy System

The government should formulate more detailed and comprehensive policies with strong operability. Improving the rigor of policies will increase the workload of policymakers but reduce possible deviations and loopholes in policy implementation. Making policies highly practical will increase the willingness of parties to meet their obligations, and more specific policy requirements will likely increase the number of willing investors. Therefore, the Chinese government should clarify the status of CCUS in policies and formulate a detailed, reliable, and operational roadmap for developing CCUS. In addition, government policies should encourage third-party access to CCUS infrastructure and prioritize open-technology or open-access CCUS projects to maximize knowledge sharing. Finally, to ensure the CCUS policy framework is effective in regional jurisdictions, the government should require provinces, cities, and autonomous regions to comply with national requirements, enact local regulations based on the actual situation, establish CCUS registration databases, and improve local supervision and management systems. In response, the Chinese government should establish non-profit organizations or think tanks related to national and provincial CCUS to further promote the formulation of policies, regulations, and standards in China.

### 6.3. Expand Government Subsidies

China’s current subsidy policy is detrimental to the long-term development of CCUS. The initial construction cost of CCUS projects is high, and insufficient government support and subsidies greatly discourage enterprises from investing in such projects. Moreover, the cost of carbon capture is also high, and existing subsidies leave companies that do pursue CCUS projects facing a long-term loss, which is likely to lead to the end of CCUS projects. To promote CCUS projects in China, the government should formulate more powerful subsidy policies. First, the amount of investment in CCUS should be increased. Similar to its approach toward developing renewable energy, the Chinese government should set up a special fund for subsidizing and providing other forms of financial support to CCUS projects. Second, to address the high cost of carbon capture, a tax exemption should be granted to enterprises engaged in this activity, thereby reducing their input costs. Meanwhile, the Chinese government should formulate relevant policies and regulations to impose a carbon tax on high-emission enterprises so as to reduce financial pressure on the government. CCUS technology has strong public-welfare benefits, and the early development of the CCUS industry depends strongly on public financial support. In particular, CCUS technology in China is in the research and development and early demonstration stage, so public finance must “transfuse” the CCUS industry and play a key role in the long-term.

### 6.4. Improve Public Awareness of CCUS

In China, public understanding of and support for industries can also play an important role in promoting their emergence. To develop a renewable energy industry, the Chinese government needs to actively promote renewable energy to the public. If the public has a great interest in new energy, enterprises will also show greater confidence in renewable energy, pour more money into the research and development of renewable energy technology, and develop renewable energy projects. This is also an important reason to rapidly develop the renewable energy industry in China. However, CCUS technology has been promoted far less by the Chinese government compared to new energy, resulting in little public understanding of CCUS technology and a lack of market interest in CCUS investment. If no measures are taken, the CCUS industry will become more marginalized in China’s future development. Therefore, the government needs to expand the promotion of CCUS technology to the public, thereby increasing public and market support for investment in the CCUS industry. First, the government should more strongly publicize the key role of CCUS technology in alleviating the climate crisis and spread awareness of the whole process of CCUS. This would give the public a comprehensive understanding of CCUS technology, thus improving public awareness of and support for CCUS. In promoting CCUS, the government should attach importance to and support CCUS, accompanied by more detailed and specific support policies to give enterprises more confidence in the long-term development of CCUS. Judging from the successful experience of China’s new energy industry, this will greatly promote market investment in CCUS, thus promoting the further development of CCUS industrialization and marketization in China.

### 6.5. Strengthen International Exchanges and Cooperation

Expanding support from international financial institutions has become a key channel to promote CCUS project construction in China. To date, China’s domestic research institutions and enterprises have participated in a series of bilateral and multilateral cooperation projects with the European Union, the United States, Australia, Italy, and other countries, as well as international organizations such as the Asian Development Bank. Moreover, China has actively participated in multilateral frameworks such as the Carbon Sequestration Leadership Forum and the Clean Energy Ministerial Conference and sought international investment to support the technical and financial development of CCUS in China.

## 7. Conclusions

CCUS technology is a key method for the Chinese government to achieve a carbon peak and carbon neutrality. The government has not yet enacted CCUS laws. Although various policies related to CCUS technology have been introduced in recent years, they are insufficient to promote the development of CCUS in China beyond the early stages, resulting in the stagnation of CCUS deployment. To address this situation, the following suggestions are proposed:

(1) *Promulgate a special law on CCUS*. China has neither enacted a department law governing CCUS nor created any effective legal framework for developing CCUS. These legal hindrances to the deployment of CCUS should be tackled by introducing a special law.
(2) *Improve the CCUS policy system*. The Chinese government needs to develop a more comprehensive CCUS policy system prioritizing systematization and operability.
(3) *Expand government financial assistance*. CCUS projects demand huge funding, and financial support from the government can effectively build enterprises’ confidence in and enthusiasm for investing in CCUS.
(4) *Strengthen the public promotion of CCUS*. The government should more strongly publicize CCUS information to improve the public’s knowledge and acceptance of CCUS technology.
(5) *Strengthen international exchanges and cooperation*. International cooperation plays a significant role in promoting the development of CCUS in China, both in terms of technology and capital. The Chinese government should thus encourage enterprises and institutions to engage in international exchanges and cooperation.

## Figures and Tables

**Table 1:** Large CCUS projects in operation worldwide [16].

Location	Project Type	CO_2_ Storage Mode	Source of CO_2_	Annual CO_2_ Capture (Million Tons)	Began Operating
Norway’s North Sea	Industrial capture	Geological sequestration	Natural gas processing	1	1996
Alberta, Canada	Industrial capture, enhanced oil recovery	Geological sequestration	Refinery hydrogen production	1	2015
Saskatchewan, Canada	Power generation and capture (after combustion), enhanced oil recovery	Part used for oil displacement; part for geological sealing	Power generation	1	2014
Alberta, Canada	Industrial capture, enhanced oil recovery	Industrial reuse of CO_2_	Oil refining	1.2–1.4	2020
Illinois, USA	Industrial capture	Geological sequestration	Ethanol production	1	2017
Texas, USA	Power generation and capture (after combustion)	Enhanced oil recovery	Power generation	1.4	2017
Kansas, USA	Industrial capture, fertilizer production	Enhanced oil recovery	Fertilizer production	1	2013
Wyoming, USA	Industrial capture	Enhanced oil recovery	Natural gas processing	1	2013
Texas, USA	Industrial capture	Enhanced oil recovery	Natural gas processing	8.4	2010
North Dakota, USA	Power generation and capture (pre-combustion)	Enhanced oil recovery	Natural gas processing	3	2000
Wyoming, USA	Industrial capture, enhanced oil recovery	Part used for oil displacement; part for geological sealing	Natural gas processing	7	1986

**Table 2:** CCUS projects with an annual catch of over 100,000 tons in China [22].

Project Name	Location	Annual CO_2_ Capture (10,000 t.a-1)	Source of CO_2_	CO_2_ Storage Mode	Began Operating
Research and Demonstration of CO_2_-EOR in Jilin Oilfield (Petrochina)	Jilin	35	Natural gas processing	EOR	2007
Shanghai Shidongkou Capture Demonstration (Huaneng Group)	Shidongkou	12	Coal-fired power plants	Commercial use	2009
CCU Demonstration in Shengli Oilfield	Shandong	40	Coal-fired power plants	EOR	2010
Shenhua Ordos New Coal-to-Oil CO_2_ Capture and Storage	Ordos	10	Coal-to-oil plant	Geological sequestration	2011
Demonstration of Capture, Utilization, and Storage in Huaneng IGCC Power Plant	Tianjin	6–10	Coal-fired power plants	EOR	2016
Xinjiang Dunhuali Carbon Dioxide Capture	Karamay	10	PSA release gas in methanol plant	EOR	2016

## Data Availability

Not applicable.
